# Molecular mechanisms underlying T cell subset imbalance and precision therapeutic targets in primary biliary cholangitis

**DOI:** 10.3389/fphar.2026.1769883

**Published:** 2026-04-22

**Authors:** Bukun Zhu, Chenyang Zhang, Jialing Ma, Zhanyang Luo, Shuyun Huang, Xingchen Yang, Liqiong Wang, Wei Zhang

**Affiliations:** Longhua Hospital Affiliated to Shanghai University of Traditional Chinese Medicine, Shanghai, China

**Keywords:** cytotoxic CD8^+^ T cells, precision immunotherapy, primary biliary cholangitis, T cell subset imbalance, Th17/Treg axis, γδ T cells

## Abstract

Primary biliary cholangitis (PBC) is a chronic autoimmune cholestatic liver disease characterized by progressive destruction of small intrahepatic bile ducts and cholestasis-associated hepatic fibrosis. Although ursodeoxycholic acid and second-line agents improve biochemical indices in many patients, approximately 10%–20% still progress to cirrhosis or require liver transplantation. This therapeutic gap reflects the bidirectional, self-amplifying interplay between intrahepatic cholestasis and immune dysregulation, while the core immunopathological mechanisms remain incompletely defined and single-effector cell-centered models are insufficient to explain bile duct–targeted injury. Within this context, T cell subset imbalance has emerged as a key conceptual framework for precision intervention. This review synthesizes current evidence on T cell subset imbalance in PBC and posits that T cell heterogeneity and network dysregulation constitute both a central pathogenic basis and a rational therapeutic target. Specifically, imbalance of the Th17/Treg ratio among CD4^+^ T cells, clonal expansion of bile duct-specific CD8^+^ T cells, and quantitative and functional abnormalities of unconventional T cell subsets — including γδ T cells, double-negative T cells, mucosal-associated invariant T cells, and invariant natural killer T cells — are closely associated with disease activity, cholangitis severity, and fibrosis progression. This review discusses how the Th17/Treg axis, cytotoxic CD8^+^ T cells, and γδ T cells interact in the pathogenesis of biliary epithelial cell injury, inflammatory microenvironment remodeling, and the vicious cycle between impaired mitophagy and immune activation. Moreover, this paper summarizes current progress and unresolved issues related to putative or emerging targeted therapeutic strategies in PBC, including IL-17 pathway inhibition, CD8^+^ T-cell-directed approaches, γδ T-cell modulation, and combination immunotherapy, most of which remain preclinical or indirectly supported in PBC. In conclusion, we present perspectives of future directions for precision immunotherapy in PBC with a focus on major gaps in knowledge such as those that exist between animal models and human disease, the impact of tissue-resident memory T cells in the longer term, and how single-cell multi-omics and spatial omics can accelerate mechanistic insights and translate these more rapidly into the clinic.

## 1 Introduction

Primary biliary cholangitis (PBC) is an autoimmune, chronic, and progressive liver disease characterized by the gradual destruction of intrahepatic small bile ducts, which leads to cholestasis and then to fibrosis or cirrhosis. The disease is driven by a breakdown in immune tolerance to bile duct-associated self-antigens (Tana and Hirschfield, 2026). PBC exhibits a broad clinical spectrum, from silent biochemical abnormalities (Kowdley et al., 2025) to nonspecific complaints like fatigue (Koc et al., 2025) or itching (Lohse and Assis, 2025). With advancing disease, patients can develop jaundice, portal hypertension, and hepatic insufficiency with a significant impact on quality of life and long-term survival (Sierra et al., 2025). Ursodeoxycholic acid (UDCA) is the standard first-line therapy; however, around 40% of patients fail to obtain a sufficient biochemical response (Tanaka, 2024) and experience worse clinical outcomes (Mandea et al., 2025). Second-line therapies, such as obeticholic acid (Li J. et al., 2025), elafibranor (Kowdley et al., 2024), and seladelpar (Hirschfield et al., 2024), are used for patients who do not respond to UDCA, with reported response rates of around 46%, 51%, and 62%, respectively. Nevertheless, 10%–20% of patients remain refractory to current regimens or progress to end-stage liver disease (Tanaka, 2024; Mandea et al., 2025; Jones et al., 2024). PBC is therefore best conceptualized as a disorder in which intrahepatic cholestasis and immune imbalance are causally interlinked and mutually reinforcing (Jones et al., 2024; Jia et al., 2022), while existing therapies have limited capacity to precisely modulate this complex immunopathological network. In this context, early identification of treatment-refractory subgroups and the development of individualized immunomodulatory strategies in combination with standard therapy represent pressing clinical priorities in PBC.

Accumulating evidence indicates that T cell subset-driven immune imbalance is central to PBC pathogenesis. Histological and immunological analyses show that portal inflammatory infiltrates are predominantly composed of CD4^+^ and CD8^+^ T cells, with concomitant enrichment of γδ T cells and other innate immune populations (Han et al., 2025; Koda et al., 2025; Shi et al., 2024). Of these, CD8^+^ cytotoxic T lymphocytes (CTLs) directly damage biliary epithelial cells through a perforin-granzyme mechanism, thus inducing bile duct destruction (Zhang et al., 2022). Meanwhile, the increase of Th17 cells and decrease of regulatory T cell (Treg) (both in peripheral and intrahepatic CD4^+^ T cells) lead to a high ratio of Th17/Treg cells, which is positively correlated with liver dysfunction and disease activities, pointing to the Th17/Treg axis as being critical for driving inflammation exacerbation and poor immunomodulation (Wang et al., 2024; Hao et al., 2019). γδ T cells, which bridge innate and adaptive immunity, are also quantitatively and functionally altered in PBC (Jang et al., 2020). Additionally, the reduced number and dysfunction of double-negative T cells may weaken their ability to regulate effector CD4^+^ T cells and cytotoxic CD8^+^ T cells, thereby promoting immune tolerance loss and disease progression (Li S. X. et al., 2019). Moreover, aberrant activation of mucosal-associated invariant T cells by microbial-derived riboflavin metabolites, recognized through a semi-invariant T cell receptor, has been implicated in PBC pathogenesis (Setsu et al., 2018), whereas imbalance among invariant natural killer T cell subsets appears to modulate the course of bile duct injury (Hebbandi Nanjundappa et al., 2024). Collectively, these findings indicate that PBC arises not from isolated dysregulation of a single T cell population, but from a spatiotemporally coordinated imbalance across multiple T cell subsets that collectively drive immune-mediated biliary injury.

Although PBC is now widely recognized as a T cell-mediated cholangiopathy, the division of labor among distinct T cell subsets across disease stages and tissue microenvironments — and the mechanisms governing their interactions — remain incompletely defined. Several critical questions therefore remain unresolved. First, how does Th17/Treg imbalance integrate with the local biliary inflammatory microenvironment, mitophagy defects, and disordered bile acid metabolism to sustain bile duct injury and drive fibrosis progression? Secondly, how do bile duct-specific CD8^+^ T cell clones, modulated by T cell receptor (TCR) signal strength, co-stimulatory and co-inhibitory pathways, and tissue-resident memory programming, constitute and maintain “hard-to-disengage” an effector pool in the biliary niche? Third, to what extent do γδ T cells act primarily as pro-inflammatory effectors versus context-dependent immunoregulatory or protective cells at various stages of the disease, and how do they functionally interact with the bile acid-farnesoid X receptor (FXR) signaling axis? Finally, one of the greatest challenges to precision medicine in PBC will be how to translate this dynamic immune landscape into meaningful diagnostic biomarkers as well as druggable targets, enabling treatment stratification and personalized immunotherapy.

Herein, we summarize recent studies aiming at providing a better understanding of the pathophysiology of altered T cell subsets as well as opportunities for targeted treatment strategies in PBC. We propose an integrated framework encompassing T cell lineage and subset features, cooperative mechanisms of bile duct injury, and key signaling pathways, and extending to biomarker development, targeted therapeutic strategies, unresolved questions, and future research priorities. Collectively, this synthesis aims to refine mechanistic understanding of PBC immunopathology and support translation toward precision clinical care.

## 2 The T cell subset imbalance in PBC

### 2.1 CD4^+^ T cell subset imbalance

#### 2.1.1 Th17/Treg axis imbalance

In PBC, disruption of the Th17/Treg balance may initiate a feed-forward loop linking biliary inflammation, impaired mitophagy, and dysregulated bile acid metabolism. Consistent with greater disease activity, the Th17/Treg ratio is significantly associated with serum alanine aminotransferase, aspartate aminotransferase, alkaline phosphatase, γ-glutamyl transferase, and total bilirubin levels (Liu et al., 2026; Mulcahy et al., 2023). Expansion of Th17 cells, together with a relative reduction in Tregs, sustains secretion of pro-inflammatory cytokines, including IL-17A/F and IL-21 (Chan et al., 2024; Cichoż-Lach et al., 2018). IL-17 acts on biliary epithelial cells and portal immune cells and, in concert with accumulated cytotoxic bile acids, activates NF-κB and mitogen-activated protein kinase signaling, thereby inducing IL-6, IL-8, and CCL20 expression. These mediators promote neutrophil and monocyte recruitment, perpetuate an inflammatory and oxidative microenvironment, and drive fibroblast activation and fibrogenesis (Fuchs et al., 2025; Prieto et al., 2021). In the setting of recurrent mitochondrial injury and defective mitophagy, reactive oxygen species (ROS), mitochondrial DNA (mtDNA), and mitochondrial autoantigens - most notably the E2 subunit of the pyruvate dehydrogenase complex (PDC-E2) - accumulate. This, in turn, enhances oxidative stress and the expression of mitochondria-derived self-antigens, thus promoting Th17 polarization and disturbing Treg homeostasis. It also promotes the senescence of biliary epithelial cells. A Senescent-associated secretory phenotype (SASP) is induced in cholangiocytes, which is accompanied by elevated levels of IL-6 and IL-8 and additionally shifts the differentiation pattern of CD4^+^ T cells towards a Th17-like profile (Prieto et al., 2021; Sasaki et al., 2018). At the same time, total bile acid concentrations rise, with the higher content in hydrophobic and cytotoxic species. These bile acids directly impair mitochondrial integrity, downregulate anion exchanger 2 expression, and reshape CD4^+^ T cell fate via signaling mediated by the FXR, Takeda G protein-coupled receptor 5 (TGR5), and the sphingosine-1-phosphate receptor-signal transducer and activator of transcription 3 (S1PR-STAT3) axis. Collectively, these pathways converge to reinforce the initiating pathogenic feed-forward loop (Kudira et al., 2025; Evangelakos et al., 2021; Hang et al., 2019; van de Graaf and Beuers, 2014).

#### 2.1.2 IL-23/IL-17 signaling axis

The IL-23/IL-17 axis is a key amplifier of T cell subset imbalance in PBC. In peripheral blood from patients with PBC, IL-23 increases Th17 cell frequency while reducing Tregs, thereby maintaining a pro-inflammatory shift in the Th17/Treg ratio (Yang et al., 2014; Qian et al., 2013). Mechanistically, IL-23 promotes Th17 expansion; Th17 cells secrete IL-17; and IL-17 further amplifies inflammatory signaling in a feed-forward loop (Cichoż-Lach et al., 2018). IL-23 is produced predominantly by dendritic cells and macrophages within inflamed, tissue-injury microenvironments. Engagement of the IL-23 receptor on Th17 cells activates the JAK2/TYK2-STAT3 pathway, sustains RORγt expression, and drives the expansion of pathogenic Th17 populations. The resulting IL-17A/F acts on biliary epithelial cells, endothelial cells, and fibroblasts to upregulate chemokines and adhesion molecules, thereby exacerbating periductal inflammation and fibrogenesis (Konstantis et al., 2025). In children with primary nephrotic syndrome (PNS), IL-23–activated γδ T cells have been reported to secrete IL-21, thereby promoting Th17 differentiation while impairing Treg stability/function, which may form a self-amplifying inflammatory feedback loop (Zhang L. et al., 2018); however, whether a similar γδ T cell–IL-21–Th17/Treg axis operates in PBC remains to be validated.

### 2.2 Bile duct-specific cytotoxic mechanisms of CD8^+^ T cells

CD8^+^ T cells constitute the principal effector population mediating biliary epithelial cell (BEC) injury in PBC, with cytotoxicity largely dependent on the perforin-granzyme pathway to achieve targeted BEC clearance (Lin et al., 2024; Zhu G. et al., 2024). Guided by chemokine gradients, CD8^+^ T cells are recruited to the liver and periductal regions, where they recognize antigenic peptide-major histocompatibility complex class I complexes on BECs and initiate cytotoxic effector programs. Perforin forms transmembrane pores in the BEC plasma membrane, permitting granzyme entry into the cytosol, activation of caspase cascades, and induction of apoptotic cell death, culminating in destruction of small bile ducts and impairment of bile flow (Lin et al., 2024; Zhu H. et al., 2024). Histopathological analyses further demonstrate that intrahepatic CD8^+^ T cells in PBC can physically intercalate into the BEC cytoplasm and exhibit increased cell size, polarized morphology, and high expression of tissue-resident markers, including E-cadherin, CD103, and CD69, consistent with a bile duct-localized Trm phenotype (Davies et al., 2024). In experimental autoimmune cholangitis, genetic ablation of CD8α or therapeutic blockade with anti-CD8α antibodies markedly attenuates immune-mediated biliary injury, providing direct evidence that CD8^+^ T cells exert highly bile duct-specific pathogenic effects (Zhu G. et al., 2024).

On this basis, multiple functional CD8^+^ T cell subsets and co-stimulatory/co-inhibitory pathways have been implicated in fine-tuning CD8^+^ T cell pathogenicity in PBC. PD-1^+^ CD8^+^ T cells undergo clonal expansion in the livers of PBC model mice; selective depletion of this population using PD-1-directed chimeric antigen receptor T cells markedly attenuates hepatic inflammation and bile duct injury, indicating that PD-1^+^ CD8^+^ T cells constitute a highly activated, tissue-destructive clonal pool (Zhang et al., 2022). CXCR6^+^ CD8^+^ T cells are also markedly enriched in the livers of patients with PBC, and their frequency correlates positively with serum alkaline phosphatase and γ-glutamyl transferase levels, autoantibody titers (antinuclear antibodies, IgG, and IgM), as well as histological inflammation grade and fibrosis stage. These associations suggest a sustained contribution of CXCR6^+^ CD8^+^ T cells to cholangiocyte-targeted injury and fibrogenesis across disease stages (Shi et al., 2024). In parallel, the balance between the co-stimulatory receptor CD226 and the co-inhibitory receptor TIGIT on CD8^+^ and CD4^+^ T cells is significantly disrupted in PBC. The proportions of CD226^+^ and TIGIT^+^ T cells are increased, and the CD226/TIGIT ratio on CD8^+^ T cells correlates positively with total bilirubin and γ-glutamyl transferase levels but inversely with serum albumin concentration and platelet count. Functionally, CD8^+^CD226^+^ T cells display enhanced effector activity compared with CD226^-^ counterparts, and pharmacological or antibody-mediated CD226 blockade suppresses their activation and proliferation (Deng et al., 2020). Collectively, these findings indicate that CD226/TIGIT disequilibrium promotes excessive CD8^+^ T cell activation and loss of immune tolerance in PBC. Accordingly, the CD226/TIGIT ratio on CD8^+^ T cells may serve as a candidate biomarker of disease activity and bile duct injury, whereas CD8^+^CD226^+^ effector subsets and their associated signaling pathways should currently be regarded as mechanistically attractive but not yet validated therapeutic targets in PBC.

### 2.3 Immunoregulatory roles of unconventional T cell subsets

Beyond conventional CD4^+^ and CD8^+^ T cells, several unconventional T cell subsets contribute substantially to immune dysregulation in PBC. γδ T cells are increased in both peripheral blood and liver tissue of patients with PBC, with a pronounced expansion of the Vδ1-dominant subset (Chen et al., 2022; Hua et al., 2016). Upon activation via their T cell receptor and co-receptors, γδ T cells rapidly secrete TNF-α, IFN-γ, IL-17 and IL-21, and related cytokines. Research indicates that Vδ1 T cells exhibit elevated production of TNF-α and IFN-γ, with heightened IFN-γ levels potentially contributing to the cytotoxic activity of CD8^+^ T lymphocytes (Hua et al., 2016). In autoimmune hepatitis (AIH), increased secretion of TNF-α and IFN-γ correlates significantly with biochemical markers of hepatocellular injury (Haas et al., 1993). Similarly, in PBC, these cytokines are implicated in the pathogenesis of hepatic inflammation and fibrosis (Li et al., 2005b; Takii et al., 2005). Notably, Vδ1 T cells preferentially accumulate in the liver and display enhanced local secretion of IFN-γ and TNF-α, thereby promoting hepatobiliary cell damage (Agrati et al., 2001; Fischer et al., 2007; Shimoda et al., 2012). Specifically, intrahepatic overexpression of IFN-γ and TNF-α drives chronic non-suppurative cholangitis and induces apoptosis of bile duct epithelial cells (Mueller et al., 2011). As a critical immunoregulatory subset bridging innate and adaptive immunity, γδ T cells demonstrate both quantitative and functional alterations in PBC patients. Through the secretion of IL-21 and IL-17, they promote Th17 cell differentiation while concurrently impairing Treg cell homeostasis — thus disrupting the balance of the T-cell subset network. These findings collectively indicate that γδ T cells actively contribute to periductal inflammatory infiltration and indirectly amplify CD4^+^ T cell-mediated injury by promoting Th17 differentiation and destabilizing regulatory T cell homeostasis. This dual mode of action sustains and reinforces cholangitic inflammation in PBC (Chen et al., 2022; Hua et al., 2016).

Double-negative T cells (DNTs, CD3^+^CD4^−^CD8^−^) are regarded as crucial immune “brakes,” capable of suppressing the proliferation of CD3^+^ T cells and playing a protective role in immune tolerance, thereby maintaining liver immune homeostasis. Research has demonstrated that DN T cells exert immunoregulatory effects in mouse liver injury models, inhibiting immune responses and protecting the liver from excessive immune attacks (Zhao et al., 2016). However, the function of DN T cells in patients with PBC is significantly impaired, with weakened inhibition of the proliferation of autologous CD4^+^ and CD8^+^ T cells, leading to the loss of their immunoregulatory function on effector T cells, disruption of the liver’s immune tolerance mechanism, and subsequent promotion of the occurrence and development of PBC. In TCR transgenic mouse models, DN T cells have been shown to highly express perforin and granzyme B, and their inhibitory function is dependent on perforin/granzyme-mediated mechanisms (Hillhouse and Lesage, 2013; Juvet and Zhang, 2012). We further confirmed that in healthy controls (HC), circulating DNT cells can strongly inhibit the proliferation of autologous CD4^+^ and CD8^+^ T cells. In contrast, the number and function of circulating DNT cells in PBC patients are significantly reduced, with weakened inhibitory capacity on self-reactive CD4^+^/CD8^+^ T cells, resulting in the continuous expansion of self-reactive clones that should be restricted, lacking an effective immune “shutdown,” thereby promoting immune attacks against bile duct-related autoantigens and becoming an important factor for the persistence of chronic inflammation (Li S. X. et al., 2019; Zhang C. et al., 2025).

Mucosa-associated invariant T (MAIT) cells serve as critical immunological mediators linking microbial cues to autoimmune pathogenesis. In patients with PBC, hepatic MAIT cell frequencies are markedly diminished, and a concomitant reduction in the proportion of circulating MAIT cells has been consistently observed (Setsu et al., 2018). Hepatic MAIT cells preferentially express the cholangiocyte-tropic chemokine receptors CCR6 and CXCR6, whereas peripheral MAIT cells retain expression of CCR6 but not CXCR6 (Jeffery et al., 2016). Furthermore, surface activation marker CD69 — as well as key cytokine receptors IL-7Rα (CD127) and IL-18Rα — are significantly downregulated on peripheral blood MAIT cells from PBC patients. Notably, genome-wide association studies (GWAS) have identified the IL7R locus as a robust genetic susceptibility factor for PBC (Nakamura et al., 2012); its encoded receptor, IL-7Rα, is functionally engaged by IL-7 — a cytokine secreted by hepatocytes and damaged liver parenchyma — that contributes to both local immune regulation and systemic immune homeostasis (Sawa et al., 2009). IL-7 signaling confers functional competence to MAIT cells, enabling MR1-independent, rapid production of pro-inflammatory cytokines such as IFN-γ and TNF-α (Tang et al., 2013). Clinically, serum IL-18 concentrations are significantly elevated in PBC patients (Yamano et al., 2000), consistent with heightened innate immune activation. Histologically, MAIT cells are constitutively enriched in the portal tracts of healthy livers — particularly in proximity to bile ducts — suggesting a physiological role in biliary immune surveillance and epithelial homeostasis, potentially mediated via direct cellular contact or paracrine signaling (Jeffery et al., 2016). Mechanistically, MAIT cells recognize microbial riboflavin-derived metabolites presented by MR1; however, dysbiosis or bacterial translocation can trigger aberrant MAIT cell activation, resulting in excessive secretion of inflammatory effectors. This pathway implicates the gut–liver axis as a plausible contributor to PBC immunopathogenesis (Setsu et al., 2018).

Invariant natural killer T (iNKT) cells play a pivotal role in immune homeostasis by modulating the balance between pro-inflammatory and regulatory responses upon recognition of lipid antigens presented by CD1d molecules. In PBC, this equilibrium is disrupted: the proportion of regulatory iNKT cells is significantly reduced, whereas pro-inflammatory iNKT subsets predominate — thereby exacerbating autoreactive responses against biliary epithelial cells, impairing peripheral immune tolerance, and accelerating bile duct injury (Hebbandi Nanjundappa et al., 2024). Biliary epithelial cells present endogenous lipid antigens to iNKT cells via CD1d (Horst et al., 2021; Schrumpf et al., 2015). Notably, Schrumpf et al. (Schrumpf et al., 2015) demonstrated downregulated CD1d expression in liver tissues — including on bile duct epithelia — of both PBC and primary sclerosing cholangitis (PSC) patients, implicating defective CD1d-mediated antigen presentation as a key mechanism underlying impaired generation or function of regulatory iNKT cells. Importantly, this CD1d deficiency persists despite elevated intrahepatic levels of cytokines known to upregulate antigen-presenting molecules (e.g., IFN-γ and TNF), suggesting a disease-specific dysregulation independent of general inflammatory cues. Consequently, the functional interplay between biliary epithelial cells and iNKT cells warrants deeper mechanistic investigation in PBC pathogenesis. Furthermore, recent clinical studies report a marked expansion of total and functionally distinct iNKT subsets in peripheral blood mononuclear cells (PBMCs) of PBC patients, which correlates positively with serum IL-23 concentrations; moreover, circulating IL-17A levels show a significant positive association with histological and biochemical markers of disease severity (Jia et al., 2022). Specifically, the frequency of IL-17A–producing iNKT cells is significantly elevated in PBC patients relative to healthy controls — a finding consistent with their potential contribution to biliary inflammation and progressive hepatic fibrosis. In advanced-stage PBC, increased expression of Fas ligand (FasL) is observed not only in infiltrating iNKT cells but also in periductal lymphocytes, indicating that Fas/FasL–mediated cytotoxicity represents a plausible effector mechanism through which activated iNKT cells directly induce biliary epithelial apoptosis and sustain chronic ductopenia (Aso-Ishimoto et al., 2014).

Taken together, γδ T cells, DN T cells, MAIT cells, and iNKT cells are involved in the PBC immunopathology through coordinated quantitative and functional shifts toward increased inflammation, disrupt immune suppression, and connect microbial signals with autoimmune activation. Therefore, the composition and functional reprogramming of these atypical T cell subsets could represent exploratory targets for immune-based stratification and precision therapy in PBC.

### 2.4 Crosstalk networks among T cell subsets

The interactions among different subsets of T cells constitute an intricate and highly regulated web which impacts on the onset and the progress, and clinical course of PBC. In particular, coordinated crosstalk between γδ T cells, the Th17/Treg axis, and IL-23/IL-17 signaling forms a feedback loop promoting immune dysregulation and accelerating disease progression.

#### 2.4.1 γδ T cells drive Th17/Treg imbalance

γδ T cells are a special type of T cell population that contributes significantly to the immune microenvironment of PBC. Secretion of IL-21 by γδ T cells facilitates Th17 polarization. IL-21 binds to its receptor, which is strongly upregulated in Th17 cells and activates JAK1/JAK3-STAT3 signaling as well as RORγt, a master transcriptional regulator of the Th17 cell lineage; this promotes expression of IL-17A/F and other inflammatory cytokines (Konstantis et al., 2025). These mediators intensify hepatic inflammation, exacerbate biliary epithelial injury, and enhance inflammatory cell infiltration, thereby accelerating bile duct damage and disease progression in PBC.

Simultaneously, IL-21 disrupts Tregs through the modification of FOXP3 acetylation — resulting in the loss of functionality and reduced capacity for suppression of Th17-mediated pathology, thus promoting a self-reinforcing cycle (feed-forward) of portal tract injury and exacerbation of disease. Overall, γδ T cell–derived IL-21 is a mechanistically plausible upstream contributor to Th17 polarization in PBC, but this axis is not yet fully validated directly in PBC.

#### 2.4.2 IL-23/IL-17 axis amplifies pathogenic Th17 immunity

The IL-23/IL-17 axis provides a key link between innate and adaptive immunity in PBC. IL-23 is produced predominantly by dendritic cells and macrophages within the injured biliary microenvironment. Engagement of IL-23 with its receptor on Th17 cells activates JAK2/TYK2-STAT3 signaling, stabilizes RORγt expression, and drives expansion of pathogenic Th17 populations (Yang et al., 2014; Qian et al., 2013). These Th17 cells secrete IL-17A/F and related cytokines that act on biliary epithelial cells, as well as hepatic endothelial and stromal cells, promoting chemokine induction, leukocyte recruitment, and fibrogenic activation. In this way, IL-23/IL-17 signaling amplifies Th17-skewed immunity and consolidates the Th17/Treg imbalance initiated by γδ T cell-derived IL-21. Overall, IL-23/IL-17 signaling appears to represent a major mechanistic node in PBC by linking innate immune activation to sustained adaptive Th17 responses and thereby driving persistent biliary inflammation and injury.

#### 2.4.3 IL-23/IL-17–CD8^+^ T cell crosstalk and immune checkpoint regulation

The IL-23/IL-17 axis also intersects with CD8^+^ T cell cytotoxic responses. IL-17 is thought to promote recruitment and activation of CD8^+^ T cells within the biliary microenvironment, whereas IL-23-driven inflammatory cues influence differentiation, maintenance, and survival of tissue-resident CD8^+^ effector cells (Jones et al., 2024). In parallel, the PD-1/PD-L1 pathway functions as a central immune checkpoint that regulates CD8^+^ T cell cytotoxicity and exhaustion, thereby shaping crosstalk among immune subsets (Mandea et al., 2025). Together, these routes create a complicated immunoregulatory circuit in which γδ T cells, Th17/Treg imbalance, IL-23/IL-17 pathway, and CD8^+^ T cell cytotoxicity interplay to induce immune-mediated biliary damage in PBC. Collectively, these interconnected pathways form an integrated immunoregulatory network in PBC that couples Th17-skewed inflammation with CD8^+^ T-cell cytotoxicity and checkpoint modulation, thereby sustaining immune-mediated biliary injury. Importantly, however, the translational readiness of these pathways is not uniform: some are supported by direct human disease-associated evidence, whereas others remain indirect, preclinical, or hypothesis-generating as pharmacologic targets in PBC.

Table 1 summarizes key T-cell populations implicated in PBC, including their characteristic cytokines or effector molecules, proposed roles in biliary inflammation and immune dysregulation, and the main sources of supporting evidence.

**TABLE 1:** Major T-cell subsets involved in PBC pathogenesis.

T-cell subset	Key cytokines/effector molecules	Proposed role in PBC	Evidence source
Th17 cells	IL-17A, IL-17F, IL-21	Promote biliary inflammation, recruit immune cells, and contribute to Th17/Treg imbalance	Human blood and liver tissue
Treg cells	IL-10, TGF-β	Maintain immune tolerance; impaired function contributes to loss of immune regulation	Human studies
CD8^+^ T cells	IFN-γ, perforin, granzyme B	Mediate cytotoxic injury to cholangiocytes and contribute to bile duct damage	Human tissue and animal models
γδ T cells	IL-17, IL-21	Amplify inflammatory responses and promote Th17 polarization	Human and experimental studies
Double-negative T cells (DNT)	Perforin, granzyme B	Suppress autoreactive T cells and maintain immune tolerance; reduced number/function in PBC	Human peripheral blood
MAIT cells	IFN-γ, TNF-α	Respond to microbial metabolites and may link gut–liver axis to immune activation	Human studies
iNKT cells	IL-4, IFN-γ, IL-17	Bridge innate and adaptive immunity; contribute to inflammatory or regulatory responses	Human and animal studies

This table summarizes key T-cell populations implicated in PBC, including their characteristic cytokines or effector molecules, proposed roles in biliary inflammation and immune dysregulation, and the main sources of supporting evidence.

Abbreviations: PBC, primary biliary cholangitis; Th, T helper cell; Treg, regulatory T cell; DNT, double-negative T cell; MAIT, mucosal-associated invariant T cell; iNKT, invariant natural killer T cell.

## 3 Multicellular collaboration and molecular basis of biliary immunoinjury in PBC

### 3.1 Synergistic mechanisms of bile duct injury

#### 3.1.1 Th17 cell-driven inflammatory microenvironment

Th17 cells are key mediators of PBC pathogenesis, secreting effector cytokines that act within the biliary microenvironment to sustain inflammation and exacerbate BEC injury (Chan et al., 2024). IL-6, a major regulator of Th17 differentiation, promotes polarization of naïve CD4^+^ T cells into Th17 lineage as well as exacerbates Th17-mediated tissue damage (Han et al., 2025). In return, IL-17 and other cytokines released from Th17 cells act on BECs to induce expression of chemokines like CXCL12 that attract and keep Th17 cells within the liver, contributing to an auto-amplifying pro-inflammatory cycle (Ye et al., 2025).

During PBC progression, Th17 cells function not only as terminal effector cells, but also as key organizers of the periductal cytokine–chemokine network, thereby sustaining a pro-inflammatory milieu that aggravates cholangiopathy and fibrosis and promotes progression toward irreversible structural remodeling. These findings support a pathogenic role for Th17-skewed inflammation in PBC. However, the pharmacologic actionability of this pathway in PBC remains variable and is discussed below according to translational maturity and strength of supporting evidence.

#### 3.1.2 Direct cytotoxicity of CD8^+^ T cells via the perforin/granzyme pathway

CD8^+^ T cells contribute to bile duct destruction by direct cytotoxic activity and are thought to be involved in PBC pathogenesis. In PBC, histopathologically CD8^+^ T cells have been found to invade BEC cytoplasms, showing an expanded, polarized morphology and high expression of tissue resident markers like E-cadherin or CD103 and CD69 (Davies et al., 2024). This “cell-in-cell” invasive pattern appears to be independent of vesicle-mediated endocytosis and thus suggests invasion by direct membrane contact (Davies et al., 2024).

From a mechanistic point of view, activated CD8^+^ T lymphocytes secrete cytotoxic granules consisting of perforin/granzymes that induce apoptosis in BECs leading to the destruction of the biliary tree (Zhang et al., 2022), while in EAC, treatment of these mice with anti-CD8α antibodies greatly reduces the biliary pathology and provides a functional proof for CD8^+^ T cell responsibility in bile duct-specific immunity-mediated injury (Zhu G. et al., 2024). Furthermore, IL-17A also leads to upregulation of PD-L1 expression on BECs implying that stimulation of the PD-1/PD-L1 pathway might act as a negative-feedback check point for limiting CD8^+^ T cell cytotoxicity in order to preserve a fine balance between tissue injury and protective control (Liaskou et al., 2018).

Taken together, these findings support a disease-relevant role for bile duct-reactive CD8^+^ T cells in mediating cytotoxic biliary injury in PBC. Nevertheless, the translational maturity of CD8^+^ T-cell-directed strategies remains heterogeneous, ranging from human disease-associated concepts to preclinical intervention models, and their therapeutic relevance should therefore be interpreted according to evidence level rather than assumed to be uniformly actionable.

#### 3.1.3 γδ T cell-mediated disruption of the biliary epithelial barrier

The quantitatively and functionally aberrant γδ T cells have been found to be significantly affected in PBC. Flow cytometry analysis reveals elevated γδ T cell frequencies in both peripheral blood and liver tissue, along with altered expression of activation and effector markers (Chen et al., 2022). In particular, circulating Vδ1^+^ γδ T cells are expanded indicating the involvement of specific γδ subsets in the disease pathogenesis (Hua et al., 2016).

Functionally, there are several ways in which γδ T cells could interfere with the biliary epithelial barrier. Firstly, a sub-set of them differentiate to become IL-17A producing γδ T17 cells, which contribute directly to the aggravation of cholangitic inflammation and BEC injury (Chen et al., 2020). Second, in hypoxic condition, γδ T cell increases the expression of programmed death ligand 1 (PD-L1) that can induce CD8^+^ T cell apoptosis by PD-1/PD-L1 signaling, thus impairing immune surveillance, which can exacerbate tissue injury (Sureshbabu et al., 2020).

Interestingly, γδ T cells may also exert context-dependent immunoregulatory functions. γδ T-cell-derived IFN-γ has been reported to inhibit Th17 differentiation; however, this potentially protective effect appears to be diminished or lost in PBC. Overall, γδ T cells in PBC appear to exhibit functionally divergent programs: γδT17 polarization and elevated PD-L1 expression are associated with biliary inflammation and immune evasion, whereas IFN-γ-mediated pathways may retain immunoregulatory and anti-fibrotic properties under physiological conditions. Taken together, these observations suggest that γδ T cells are biologically relevant in PBC, but their marked functional plasticity means that γδ T-cell-targeted pharmacology remains largely exploratory at present. Accordingly, any therapeutic interpretation should be framed according to microenvironmental context, disease stage, and relative translational maturity.

#### 3.1.4 Vicious cycle between mitophagy and immune activation

Mitochondrial dysfunction and aberrant immune cell activation may establish a self-amplifying pathological loop. In PBC, accumulating evidence indicates impaired mitophagy in cholangiocytes, leading to the accumulation of dysfunctional mitochondria and subsequent release or exposure of mitochondrial damage-associated molecular patterns (DAMPs). These DAMPs can activate intrahepatic innate immune cells and drive the production of pro-inflammatory cytokines (Han et al., 2022). Although direct detection of mitochondrial stress signals specifically within the bile duct microenvironment remains to be fully characterized, the “mitochondrial stress–inflammatory amplification” axis offers a mechanistically plausible framework for future investigations. Importantly, the canonical mitochondrial autoantigen subunit in PBC should be regarded not as a conventional DAMP, but rather as a critical antigenic target that initiates and sustains adaptive immune recognition. We propose that, under conditions of cholangiocyte mitochondrial stress, PDC-E2–mediated antigen presentation and DAMP-triggered innate immune signaling may be spatiotemporally coordinated — thereby synergistically enhancing both inflammatory and autoimmune responses.

Within the inflammatory microenvironment, T cell responses may undergo further amplification. Accumulating evidence indicates that interleukin-15 (IL-15), via IL-15 receptor alpha (IL-15Rα)-mediated trans-presentation, sustains and activates Trm, including both CD8^+^ Trm and γδ Trm subsets, thereby enhancing the production of pro-inflammatory cytokines such as IFN-γ (Ma et al., 2022). Building upon these findings, we propose a mechanistic working hypothesis: persistent Trm cell activation leads to sustained release of cytotoxic effector molecules — including granzymes and perforin — which exacerbate cellular stress in bile duct epithelial cells and induce mitochondrial dysfunction. This cascade may establish a self-perpetuating pathogenic loop characterized by “mitochondrial stress/injury → immune activation → bile duct injury” (Ma et al., 2022; Zhang et al., 2022). Critically, immune activation within this loop may function not merely as an amplifier of injury signals but also as a central regulatory node driving disease chronicity. Consequently, therapeutic strategies targeting the IL-15–Trm axis, the granzyme/perforin-mediated cytotoxic pathway, and interventions that promote mitophagy or restore mitochondrial homeostasis hold promise for concurrently attenuating both the origin of injury signals and their downstream amplification. Such approaches may represent a mechanistically plausible therapeutic direction, although whether they can alter progression in PBC remains to be established.

### 3.2 Dissection of molecular regulatory pathways

#### 3.2.1 Regulation of Th17/Treg differentiation by the PPARγ/NF-κB pathway

Peroxisome proliferator-activated receptor γ (PPARγ) is a nuclear receptor that primarily regulates the Th17/Treg balance by inhibiting NF-κB signaling. In PBC, activation of PPARγ decreases the NF-κB-dependent inflammatory response and ameliorates liver damage (Mao et al., 2025; Teshima et al., 2021), whereas PPARγ deficiency increases NF-κB activity, increases production of pro-inflammatory cytokines, promotes Th17 differentiation, and impairs the function of regulatory T cells (Zhang W. et al., 2025). Furthermore, PPARγ agonists attenuate Toll-like receptor (TLR)-NF-κB signaling via downregulation of TLR2, TLR4, and TLR5, thereby further restricting the release of inflammatory cytokines (Mao et al., 2025).

Taken together, these results suggest that the PPARγ-NF-κB axis may play an important role in immune imbalance in PBC; accordingly, PPARγ is a mechanistically plausible candidate regulator of Th17/Treg balance, although its value as a therapeutic target is not yet validated directly in PBC (Ding et al., 2018).

#### 3.2.2 TCR signal strength and CD8^+^ cytotoxic effector function

The strength of stimulation through the TCR is an important determinant of CD8^+^ T cell cytoxicity. TCR stimulation in PBC upregulates N-Ras, which leads to secretion of proinflammatory mediators (Nakagawa et al., 2019). Intrahepatic CD8^+^ T cells are activated by recognition of bile duct - associated autoantigens (e.g., PDC-E2), which leads them to differentiate in CTLs able to directly damage the bile duct epithelium (Li Q. et al., 2025; Yi et al., 2020).

In addition, programmed cell PD-1 pathway is involved in regulation of CD8^+^ T cell function; aberrant PD-1 expression may disrupt peripheral tolerance and promote CD8^+^ T cell cytotoxicity in PBC (Zhang R. et al., 2018). Together, these findings highlight the importance of TCR signal strength — and its interaction with co-inhibitory pathways — as a crucial factor in CD8^+^ T cell-mediated bile duct damage.

#### 3.2.3 Regulation of γδ T cell activation by bile acid metabolites via FXR

Bile acid metabolites modulate γδ T cells activation and function through the FXR. Activation of FXR suppresses NF-κB signaling and reduces production of pro-inflammatory cytokines, including TNF-α and IL-1β, thereby attenuating hepatic inflammation (Shi et al., 2024; Kobayashi et al., 2024; Wang et al., 2021). In patients with PBC, quantitative and functional abnormalities of γδ T cells are closely associated with dysregulated bile acid metabolism (Deng et al., 2020; Vela-Ojeda et al., 2021).

Bile acid metabolites, including UDCA, can bind the ligand-binding domain of FXR (e.g., at residues Glu467, Ile317, and Leu464), promote formation of the FXR/SRC-1 coactivator complex, and regulate downstream target genes such as BSEP, MRP2, and SHP, thereby influencing γδ T cell activation (Shi et al., 2024). In parallel, FXR directly represses the NFKB1 promoter, inhibiting NF-κB signal transduction and attenuating γδ T cell-mediated inflammatory responses (Shi et al., 2024; Kobayashi et al., 2024). Collectively, these findings indicate that the bile acid-FXR axis not only governs cholestatic metabolic homeostasis but also modulates γδ T cell activation states, providing a mechanistic link between metabolic disturbance and immune dysregulation in PBC.

**FIGURE 1:**
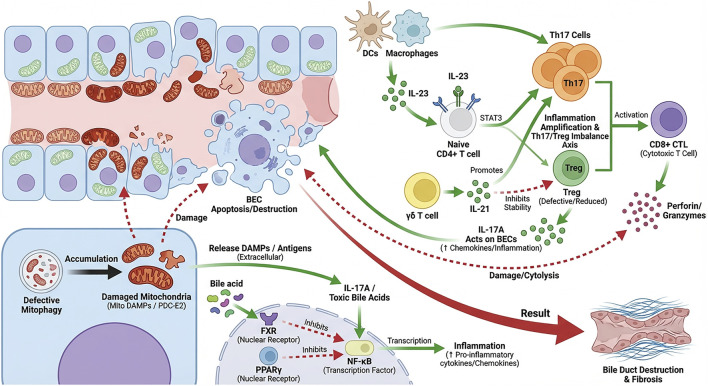
Schematic illustration of the vicious cycle underlying bile duct injury in PBC. This figure illustrates that immune disturbance, metabolism disorder and BEC damage form a self-reinforcing loop in PBC. Central to all these processes is imbalanced T cell subsets, characterized by a high Th17/Treg ratio that can be exacerbated by γδ T cells. Dendritic cells (DCs) and macrophages (Mφ) secrete IL-23, driving STAT3-dependent Th17 differentiation/expansion. γδ T cells also exert an upstream effect through the secretion of IL-21 that promotes Th17 polarization while promoting the instability of Tregs. IL-17A from Th17s affects BECs, which induces release of pro-inflammatory cytokines and chemokines that recruit immune-cells. BEC injury occurs mainly via CTLs using perforin-granzyme pathway, leading to the apoptosis of BEC and loss of bile ducts. This immune damage is amplified by another metabolic feedback loop: defective BEC mitophagy results in accumulation and secretion of mitochondrial DAMPs (e.g., PDC-E2), inducing the activation of innate immunity and promoting a Th17 response; while granzymes released from CTLs aggravate the mitochondrial injury in BECs. As a counter-regulatory mechanism, bile acid-activated FXR and Peroxisome proliferator-activated receptor γ (PPARγ) signaling converge to suppress NF-κB-dependent inflammatory transcription, reducing inflammation and promoting restoration of T cell subset balance. Although not depicted as a separate node in the central schematic, tissue-resident memory T cells (Trm) are discussed in Section 5.3 as an additional mechanism contributing to chronicity, relapse, and long-term immune persistence in PBC. Abbreviations: BEC, biliary epithelial cell; CTLs, cytotoxic T lymphocytes; DAMPs, damage-associated molecular patterns; FXR, farnesoid X receptor; PPARγ, peroxisome proliferator-activated receptor γ; TGR5, Takesda G protein-coupled receptor 5; SASP, Senescent-associated secretory phenotype; Trm, tissue-resident memory T cells.

Taken together, these findings propose a unified model where T cell subset imbalance, mitochondrial stress, and bile acid-FXR signaling work together to amplify bile duct injury (Figure 1).

## 4 Immunotherapy for PBC: biomarkers and targeted strategies

### 4.1 Potential of diagnostic biomarker development

#### 4.1.1 Peripheral Th17/Treg ratio as an indicator of disease activity

Abnormal peripheral Th17/Treg balance is a prominent immunological feature in patients with PBC. Flow cytometric analyses have shown increased frequencies and absolute numbers of peripheral blood Th17 cells, together with an elevated Th17/Treg ratio (An et al., 2019). This altered ratio is closely associated with disease activity and correlates positively with serum alanine aminotransferase (ALT), aspartate aminotransferase (AST), alkaline phosphatase (ALP), γ-glutamyltransferase (GGT), and total bilirubin (TBil) levels (Truchetet et al., 2023). Moreover, the association between the Th17/CD4^+^ Treg ratio and inflammatory disease activity in other immune-mediated conditions suggests that this metric may serve primarily as a descriptive biomarker of immune activation in PBC (An et al., 2019). In addition, because low-dose IL-2 treatment decreases the Th17/CD4^+^ Treg ratio, this parameter may have potential utility as a treatment-monitoring biomarker; however, its predictive value and clinical translatability in PBC still require prospective validation.

#### 4.1.2 Candidate predictive value of bile duct-specific CD8^+^ T-cell clones

CD8^+^ T cells play a crucial role in biliary epithelial cell injury in PBC, and defined CD8^+^ T-cell subsets may therefore serve as mechanistically informative biomarkers for immune stratification. CXCR6^+^ CD8^+^ T cells are highly enriched in liver tissues from PBC patients, with their number being proportional to the degree of cholangiopathy (Shi et al., 2024). Furthermore, single-cell RNA sequencing shows that intrahepatic memory CD8^+^ T cells recognizes immunodominant epitopes of pyruvate dehydrogenase complex E2 subunit, directly mediating bile duct-specific immune attacks (Ye et al., 2023; Peng B. et al., 2025). In keeping with these results, CD8^+^ T cells induce BEC apoptosis via the perforin-granzyme axis, a model very similar to the histopathology seen in the destruction of bile ducts in PBC (Ye et al., 2023).

Furthermore, the percentage of TIGIT-expressing CD8^+^ T cells was significantly elevated in the peripheral blood of PBC patients than healthy controls, suggesting that checkpoint-defined, aberrantly activated CD8^+^ T cell clones can be used as an early marker of immune activation (Long et al., 2017). Collectively, these observations suggest that bile duct-specific and immune checkpoint-defined CD8^+^ T-cell subsets are promising candidate predictive biomarkers for disease stratification and possibly therapeutic response in PBC. However, their clinical utility for early diagnosis, progression prediction, and treatment monitoring remains to be established in prospective validation studies.

#### 4.1.3 Tissue-specific expression profiles of γδ T cell surface markers

γδ T cells in PBC display distinct tissue distribution patterns and surface phenotypes. Flow cytometric and immunohistochemical analyses demonstrate that γδ T cell frequencies are significantly higher in liver tissue than in peripheral blood, with preferential accumulation in periductal regions within portal inflammatory infiltrates (Jiao et al., 2025). These intrahepatic γδ T cells predominantly express Vδ1-type T cell receptors, whereas Vδ2 subsets are relatively enriched in healthy controls (Pu et al., 2025).

Functionally, expression of the activation marker HLA-DR on γδ T cells is associated with inflammatory activity. In Crohn’s disease, for example, the proportion of HLA-DR^+^ CD8^+^ γδ T cells has been proposed as a marker of intestinal inflammation (Ayan et al., 2023). In PBC, γδ T cells express high levels of cytotoxic mediators, including granzyme B and perforin, and directly contribute to disruption of the biliary epithelial barrier (Jiao et al., 2025). Accordingly, inter-individual differences in the expression of activation and cytotoxic markers on γδ T cells across disease stages may provide a basis for descriptive immune stratification in PBC and may have future prognostic relevance, although their predictive performance and routine clinical translatability remain uncertain (Ayan et al., 2023).

#### 4.1.4 Translational framework for biomarker implementation in PBC

Although several candidate biomarkers have been identified in PBC, their clinical utility depends on a systematic, clinically oriented translational framework rather than isolated discoveries. At a practical level, these candidate biomarkers can be viewed across three translational tiers: descriptive biomarkers that reflect immune state or disease biology, predictive biomarkers that may help stratify progression risk or treatment response, and clinically translatable biomarkers that are feasible, reproducible, and potentially implementable in routine practice. One useful way to operationalize this framework is to classify biomarkers according to intended use: (1) early diagnosis and risk stratification, (2) assessment of immune activity and disease status, (3) prediction of progression or fibrosis-related outcomes, and (4) prediction and monitoring of treatment response. Within this framework, biomarkers linked to γδ T-cell activation, Th17/Treg imbalance, IL-23/IL-17 signaling abnormalities, or altered CD8^+^ T-cell cytotoxicity/exhaustion are currently best regarded as descriptive or mechanistically informative markers for immune phenotyping and longitudinal monitoring, whereas only a subset may eventually prove predictive or clinically translatable. Major barriers to translation include marked clinical and immunologic heterogeneity in PBC, limited generalizability of single biomarkers, and the lack of prospective multicenter validation. A further important challenge is the marked inter-individual variability in clinical response to current therapies, which complicates biomarker validation, treatment stratification, and assessment of immune-targeted interventions in PBC. Additional obstacles include nonuniform sample collection, assay platforms, analytical pipelines, and endpoint definitions, as well as technical issues such as reproducibility, inter-laboratory variability, cut-off determination, and cost-effectiveness. Peripheral blood biomarkers are accessible but may not fully capture biliary tissue immune events, whereas tissue-derived markers are more pathologically relevant but less feasible for routine use.

These limitations also highlight opportunities. Composite biomarker models integrating soluble mediators, immune-cell features, and conventional clinical parameters (e.g., biochemistry, imaging, and histology when available) may improve diagnostic and prognostic performance. Longitudinal sampling and integration with patient stratification algorithms and multi-omics data may further support the future development of more clinically translatable precision monitoring strategies in PBC.

### 4.2 Advances in targeted therapeutic strategies

#### 4.2.1 Clinical trial evidence of IL-17 inhibitors (such as secukinumab)

Th17 cells and their signature cytokine IL-17A play a pivotal role in the pathogenesis of PBC. In animal models, Th17 cells promote establishment of a periductal inflammatory microenvironment primarily through IL-17A secretion (Han et al., 2020). A clinical study in patients with myasthenia gravis has demonstrated that RORγ inhibitors significantly reduce the frequency of Th17 cells, with particularly pronounced suppression of the pathogenic Th17 subset co-expressing IFN-γ and IL-17 (Yi et al., 2020). Despite these advances, clinical evidence for IL-17-targeted therapy in PBC remains limited. IL-17 inhibitors such as secukinumab have been associated with a reduced risk of disease progression in psoriatic arthritis (Cho et al., 2025). However, their efficacy and safety in PBC have not been systematically evaluated. Moreover, IL-17 may exert context-dependent and potentially protective functions during bile duct injury (Yuan et al., 2023; González et al., 2022), raising concern that non-selective IL-17 blockade in cholangiopathies could lead to unintended adverse effects. Altogether, these findings suggest that targeting the Th17/IL-17 axis is mechanistically plausible in PBC, but this strategy is not yet validated directly in PBC and currently remains an exploratory therapeutic concept. In addition, because IL-17 contributes to mucosal and epithelial host defense, broad IL-17 blockade may increase susceptibility to infection and may also interfere with protective immune functions in the biliary microenvironment. These safety considerations further support a cautious, biomarker-guided approach to IL-17-targeted therapy in PBC, rather than non-selective pathway suppression.

#### 4.2.2 Balancing safety and efficacy in CD8^+^ T cell-depleting therapies

Cytotoxic CD8^+^ T cells play an important effector role in PBC by mediating biliary epithelial cell injury through the perforin–granzyme pathway and thereby promoting bile duct damage (Ding et al., 2018; Yeung et al., 2025). In autoimmune cholangitis models, treatment with anti-CD8α antibodies reduces biliary immunopathology, providing preclinical support for CD8^+^ T-cell-directed strategies; however, such approaches have not yet been validated directly in patients with PBC (Ding et al., 2018). Similarly, peripheral CD8^+^ T cells isolated from patients with PBC display increased expression of exhaustion markers, including PD-1, consistent with chronic antigen exposure and functional dysregulation (Zhu G. et al., 2024). However, sustained or excessive depletion of CD8^+^ T cells raises substantial safety concerns. Beyond increasing susceptibility to infection, such strategies may impair antiviral immunity and weaken tumor immune surveillance. Moreover, broad depletion may produce off-target immunosuppressive effects by compromising protective cytotoxic immune functions that are not restricted to pathogenic biliary niches. Consequently, pathway-level modulation has emerged as a potential alternative to global depletion. In animal models, the JAK1/2 inhibitor ruxolitinib attenuates interferon-γ signaling, reduces CD8^+^ T-cell infiltration, and ameliorates tissue inflammation (Truchetet et al., 2023). Taken together, these findings suggest that partial functional reprogramming or selective targeting of pathogenic CD8^+^ T-cell subsets may be mechanistically plausible and may offer a more favorable safety profile than global depletion, although the efficacy and safety of such approaches are currently supported primarily by preclinical or indirect evidence.

#### 4.2.3 Emerging applications of γδ T cell modulators

γδ T cells contribute to PBC pathogenesis through non-classical immunoregulatory mechanisms. Studies have shown a significant expansion of γδ T cells in the livers of patients with PBC (Li S. X. et al., 2019). Bruton’s tyrosine kinase (BTK) inhibitors, such as ibrutinib, have been reported to modulate signaling pathways relevant to T-cell activation; in patients with chronic lymphocytic leukemia, ibrutinib treatment markedly reduces cutaneous IL-17A levels and the frequency of IL-17A^+^ γδ T cells (Nadeem et al., 2020). Mechanistically, BTK-associated phosphorylation of phospholipase Cγ1 has been implicated in lymphocyte activation, whereas the selective BTK inhibitor acalabrutinib suppresses T-cell proliferation (Xia et al., 2020). Although direct evidence in PBC remains limited, these observations indirectly support the concept that BTK inhibition might attenuate pathogenic γδ T-cell activation and IL-17A production. In the context of PBC, BTK inhibitors should therefore be regarded as hypothesis-generating, mechanistically plausible candidates rather than validated therapeutic options, and they primarily warrant further preclinical investigation. An additional safety concern is that indiscriminate γδ T-cell targeting may suppress not only pathogenic inflammatory programs but also potentially beneficial immunoregulatory or tissue-protective functions. This functional plasticity argues for context-dependent and stage-specific therapeutic interpretation rather than broad suppression of the γδ T-cell compartment.

#### 4.2.4 Optimization of combination immunomodulatory regimens

Given the multi pathway pathogenesis of PBC, rational combination immunomodulation represents an important direction for therapeutic optimization. In PBC mouse models, low-dose interleukin-2 (IL-2) increases regulatory T cells, restores the Th17/Treg balance, and reduces liver injury (Han et al., 2022). Preclinical studies also show that nanoparticle-encapsulated rapamycin (ImmTOR) promotes a tolerogenic hepatic immune microenvironment and inhibits autoreactive T-cell responses (Marzorati et al., 2016). Designing combination therapies requires careful consideration of several factors: (1) the timing and intensity of IL-17-targeted therapy and CD8^+^ T-cell depletion or functional reprogramming, in order to avoid excessive immunosuppression; (2) the mechanistic complementarity between Bruton’s tyrosine kinase inhibitors and PPARγ agonists, which may support coordinated modulation of T-cell activation, NF-κB-related inflammatory signaling, and Th17/Treg balance (Xiao et al., 2025); and (3) the potential synergy between tolerogenic agents such as ImmTOR and first-line ursodeoxycholic acid, combining immune modulation with correction of cholestatic injury (Marzorati et al., 2016). Overall, iterative refinement of combination regimens — guided by immune phenotyping and dynamic biomarker monitoring — may provide a conceptual framework for future precision, multitarget immunotherapy in PBC, although this approach remains largely preclinical and has not yet been validated directly in PBC. From a safety perspective, combination regimens must also be evaluated for cumulative immunosuppressive burden, including increased infection risk, impairment of tumor immune surveillance, and unintended suppression of protective immune circuits. These considerations further support the development of low-intensity, reversible, and biomarker-guided combination strategies in PBC.

## 5 Current challenges and future directions

From a translational perspective, the immune pathways and candidate interventions discussed in this review should not be interpreted as being at the same stage of development. Some are supported by relatively stronger clinical or human disease-associated evidence, whereas others remain indirect, preclinical, or primarily hypothesis-generating in PBC. To improve structural clarity for a translational readership, the following discussion emphasizes relative translational maturity and strength of supporting evidence rather than treating all candidate targets as equally actionable.

### 5.1 Drug development

Multiple therapeutic strategies targeting the Th17/IL-17 axis, cytotoxic CD8^+^ T cells, and γδ T cells have shown efficacy in animal models or in other immune-mediated diseases; however, in PBC, these strategies differ substantially in evidentiary strength and pharmacologic maturity. Broadly, they may be grouped into three translational categories: (1) relatively more mature or clinically linked pathways, such as bile acid/FXR-related modulation with immunologic relevance; (2) emerging immune targets supported by human disease-associated but still indirect evidence, including the IL-23/IL-17 axis, Th17/Treg rebalance, and selected co-signaling pathways; and (3) largely preclinical or hypothesis-generating approaches, such as selective CD8^+^ T-cell targeting, BTK-related modulation of γδ T-cell responses, and several tolerance-restoring or combinatorial strategies. This hierarchy is important for avoiding overinterpretation and for aligning mechanistic plausibility with realistic translational readiness in PBC. These include IL-17 and RORγt inhibitors (Han et al., 2020; Cho et al., 2025; Yuan et al., 2023; González et al., 2022), anti-CD8α antibodies and JAK1/2 inhibitors, and Bruton’s tyrosine kinase inhibitors as modulators of γδ T cell function; most of these approaches are not yet validated directly in PBC (Li Y. et al., 2019; Nadeem et al., 2020; Xia et al., 2020). In addition, combination approaches — such as low-dose interleukin-2 with nanoparticle-encapsulated rapamycin (ImmTOR) — have partially corrected Th17/Treg imbalance and attenuated liver injury in murine models, providing preclinical proof-of-concept rather than disease-specific clinical validation in PBC (Han et al., 2022; Marzorati et al., 2016; Xiao et al., 2025).

However, high-quality clinical evidence in patients with PBC remains limited. A central challenge lies in suppressing pathogenic T-cell responses without compromising host defense against infection, weakening tumor immune surveillance, or producing off-target immunosuppressive effects in protective immune compartments. In particular, the context-dependent protective roles of IL-17 in bile duct injury (Yuan et al., 2023; González et al., 2022) and the indispensable antiviral and antitumor functions of CD8^+^ T cells argue against intensive single-axis immunosuppression. Instead, a multitarget, low-intensity, reversible, and biomarker-guided immunomodulatory strategy may be more clinically feasible in PBC. Accordingly, future trials should incorporate immune endpoints — such as the Th17/Treg ratio and the frequencies of CXCR6^+^ or CD226^+^ CD8^+^ T cells — alongside conventional biochemical markers to enable mechanism-informed therapeutic development in PBC (Han et al., 2022; Yi et al., 2020; Han et al., 2020; Cho et al., 2025; Yuan et al., 2023; González et al., 2022; Yeung et al., 2025; Zhu et al., 2024; Li Y. et al., 2019; Nadeem et al., 2020; Xia et al., 2020; Marzorati et al., 2016; Xiao et al., 2025).

### 5.2 Discrepancies between animal models and human disease

Although animal models have yielded valuable insights into the immunopathogenesis of PBC, no single model fully recapitulates the clinical, histopathological, and immunological complexity of the human disease. Established models — including Il12b/Il2ra double-knockout mice — partially reproduce key features such as autoimmune cholangitis, anti-mitochondrial antibody (AMA) production, and periportal fibrosis; however, substantial interspecies discrepancies persist in immune cell composition, bile acid metabolism, and gut–liver axis dynamics (Sorcini et al., 2023). For example, anti-CD8α antibody treatment ameliorates cholangiocyte-directed immune injury in murine models, yet its safety and therapeutic efficacy in human PBC remain unestablished due to insufficient clinical evidence (Sorcini et al., 2023). Furthermore, critical differences exist in the antigen specificity of cholangiocyte-reactive T cells and the diversity and clonal architecture of the TCR repertoire (Yamashita et al., 2023; Zhang W. et al., 2018). Collectively, these limitations underscore the need for cautious interpretation when translating mechanistic findings from individual animal models to clinical therapeutic strategies.

Current preclinical models typically recapitulate only isolated pathological dimensions — such as cholangitis-like inflammation, ductular damage, AMA-associated autoimmunity, or fibrotic remodeling — yet fail to encompass the full clinical–pathological spectrum, protracted natural history, and marked interindividual heterogeneity characteristic of human PBC (Wang et al., 2023). A fundamental constraint arises from evolutionary divergence in immune system organization and signaling networks, including imbalances in T-cell subset distribution (e.g., γδ T cells, Th17, Treg), dysregulated cytokine axes (e.g., IL-23/IL-17 amplification), altered antigen presentation pathways, and divergent immune checkpoint expression profiles. These discrepancies may compromise the fidelity of mechanistic inferences — particularly regarding CD8^+^ T-cell cytotoxicity, exhaustion phenotypes, and regulatory network dysfunction (Zhu H. et al., 2024). Moreover, many murine models depend on genetic ablation or exogenous immune perturbation, resulting in accelerated, non-physiological inflammatory trajectories that poorly mirror the indolent, stage-dependent progression observed in patients. In addition, the use of inbred strains overlooks clinically relevant variables — including sex-biased immune regulation, polygenic susceptibility, environmental modulators (e.g., microbiota, xenobiotics), and tissue-specific microenvironmental cues (De Jong, 2024). To improve translational relevance, future investigations should adopt a multimodal integrative approach: combining humanized mouse models, immune–liver chimeric systems, patient-derived bile duct and liver organoids, and microphysiological “liver–bile duct–immune cell” organ-on-chip platforms (Rezvani, 2025; Mehta et al., 2024). Critically, these systems must be anchored to multi-omics profiling data from well-phenotyped PBC cohorts to enable simultaneous modeling of core pathogenic axes — including cholestasis, T-cell dysregulation, and mitochondrial stress — within a unified, physiologically contextualized framework (Peng Z. et al., 2025). Such an integrated strategy will strengthen mechanistic validation, enhance predictive accuracy in therapeutic target identification, and increase the clinical extrapolability of preclinical drug evaluations (Sorcini et al., 2023; Yamashita et al., 2023; Zhang L. et al., 2018).

### 5.3 Long-term impact of tissue-resident memory T cells

The periductal CD8^+^ Trm, or the γδ Trm population is now thought to be “immune memory reservoirs” which maintains chronic cholangitis and can also contribute to relapse in PBC. However, their clonal dynamics, functional plasticity, and interactions with the bile acid-FXR signaling and mitophagy is poorly understood (Long et al., 2025; Li et al., 2021; Vural et al., 2021).

Multi-omics methods - such as single-cell transcriptomics, TCR sequencing, epigenomics or spatial transcriptomics - could help to elucidate the lineage relationship of Trm and to map out cell-cell interactions. However, they are accompanied with great analysis challenges due to the scale and complexity of the high dimensional data and the necessity of an efficient crossmodal integration, that is becoming more and more reliant upon deep learning algorithms such as those based around the graph attention network (GAT) architecture (Lv et al., 2024; Adossa et al., 2021). In addition, the spatial resolution and throughput of existing platforms is not yet sufficient to fully capture interactions between T cells, cholangiocytes, and fibroblasts in this confined periductal niche (Xie et al., 2023; Zheng, 2023). Finally, identifying truly druggable targets among large candidate lists demands integration with functional validation, including CRISPR-based screening techniques (Guan and Quek, 2025; Bode et al., 2021; Abbas and Wierda, 2021).

A central challenge for future precision immunotherapy in PBC will therefore be how to selectively remodel pathogenic Trm populations while preserving essential intrahepatic antiviral and antitumor immune surveillance (Long et al., 2025; Li et al., 2021; Vural et al., 2021; Lv et al., 2024; Adossa et al., 2021; Xie et al., 2023; Zheng, 2023; Guan and Quek, 2025; Bode et al., 2021; Abbas and Wierda, 2021).

### 5.4 Precision medicine pathways for individualized immune intervention

PBC has high levels of genetic and immunological heterogeneity. Variation in HLA and non-HLA risk loci, innate susceptibility genes within cholangiocytes,and the genes that regulate bile acid synthesis and degradation define different immunophenotypes and clinical outcomes (Wang et al., 2022; Xiang et al., 2021; Tanaka et al., 2018). Under these conditions, uniform immunosuppressive strategies are unlikely to be universally effective. A more promising strategy would be to develop a unified stratification scheme linking clinical features with immune/molecular phenotypes for individualized treatment decisions.

In a clinical setting, peripheral Th17/Treg ratios, bile duct-reactive CD8^+^ T cell clone prevalence, γδ T cell activation status, and bile acid-FXR signaling profiles might also be incorporated in risk assessment and therapeutic algorithms. In research, machine learning can be used in combination of multi-omics profiles and longitudinal clinical data for the generation of composite risk scores and prediction of treatment response, enabling adaptive, treat-to-target immune management approaches akin to what is applied for rheumatology (Wang et al., 2022; Xiang et al., 2021; Tanaka et al., 2018).

Collectively, this approach may enable matching different PBC immune endotypes to tailored sets of targets and treatment intensity, builds on existing treatment options such as UDCA or obeticholic acid (OCA). Such a strategy will speed up transition from empiric therapy towards personalized immunotherapy.

From the perspective of translational medicine, systematically integrating the immune regulatory pathways into a framework based on disease staging and molecular subtypes is expected to facilitate the construction of subtype-specific precision treatment strategies for PBC. In the early stage of the disease, the pathological features mainly manifest as the activation of the innate immune system, including enhanced functions of dendritic cells and macrophages, upregulated stress signals in bile duct epithelial cells, and the abnormal antigen presentation process mediated thereby. As the disease progresses, the adaptive immune response gradually takes the leading role, specifically reflected in: B cell activation and plasmablast expansion driven by the T follicular helper cells (Tfh) - interleukin-21 (IL-21) axis (Li et al., 2015; Cargill et al., 2019); imbalance of autoreactive T cell responses, such as the dysregulation of Th17/Treg ratio and enhanced infiltration of CD8^+^ cytotoxic T lymphocytes; in addition, the senescence of bile duct epithelial cells and related bile duct responses can reshape the local microenvironment of the biliary tract, continuously promoting the maintenance of chronic inflammation. Analyzing these multi-level and dynamically evolving immune events within a unified staging-subtype integration framework may help identify immune biomarkers with potential clinical predictive value and therapeutic relevance, thereby informing the future development of a more precise immune-based treatment framework for PBC.

The clinical translation of precision immunology in PBC necessitates the systematic integration of multi-omics data, well-characterized longitudinal clinical cohorts, and rigorously standardized biomarker validation frameworks. In particular, the synergistic application of single-cell and spatial omics technologies — integrated with deeply phenotyped clinical datasets — holds promise for delineating stage-specific immune perturbations, such as dysregulated T-cell networks encompassing γδ T cells, MAIT cells, iNKT cells, and cytotoxic CD8^+^ T cells. These integrative strategies are instrumental in advancing the development of clinically actionable biomarker panels to support disease stratification, therapeutic decision-making, and longitudinal treatment monitoring. Concurrently, robust ethical governance must be embedded throughout the research and implementation pipeline — including responsible stewardship of large-scale patient-derived data, stringent privacy safeguards, and proactive measures to ensure equitable access to novel diagnostic and prognostic tools. Furthermore, fostering sustained, informed patient engagement in longitudinal studies is essential to align research priorities with patient-centered outcomes and to ensure that advances in precision immunology yield tangible, equitable clinical benefits for individuals living with PBC.

### 5.5 Single-cell and spatial omics in PBC research

Recent advances in single-cell multi-omics and spatial omics technologies provide powerful tools for dissecting the complex immune landscapes that underlie PBC. Single-cell RNA sequencing (scRNA-seq) enables high-resolution characterization of cellular heterogeneity, differentiation states, and intercellular signaling networks within hepatic and immune cell populations, thereby revealing disease-relevant subsets and pathogenic pathways in autoimmune liver diseases, including PBC and PSC (Jin et al., 2024). When integrated with spatial transcriptomics, these approaches allow molecular profiles to be mapped back to their native tissue context, helping to define how specific cell types interact within the biliary microenvironment and contribute to inflammation and fibrosis (Miyamoto et al., 2025). Spatial omics methods such as deterministic barcoding in tissue (DBiT-seq) further extend this framework by enabling simultaneous spatial profiling of mRNA and protein within intact tissue architecture, thus supporting multi-omic analysis of immune cell neighborhoods and signaling niches (Liu et al., 2020). These integrative approaches not only elucidate the spatiotemporal dynamics of T-cell subsets and their interactions with immune and stromal cells, but may also facilitate a more structured ranking of candidate biomarkers and therapeutic targets according to their descriptive, predictive, and translational relevance. As scRNA-seq and spatial omics become more widely applied in liver immunology and autoimmune disease research, they are likely to refine mechanistic understanding of PBC and support stage-, endotype-, and evidence-oriented prioritization of future intervention strategies.

Table 2 summarizes the major immune pathways implicated in PBC, together with representative biomarkers/readouts, potential therapeutic angles, principal sources and levels of supporting evidence, relative translational maturity, and key practical considerations for endotype- and stage-oriented strategies.

**TABLE 2:** Key immune axes in PBC, representative biomarkers, strength of supporting evidence, and relative translational maturity.

Immune axis/pathway	Representative biomarkers/readouts	Potential therapeutic angle	Evidence source/level	Relative translational maturity	Key translational notes
Th17/Treg imbalance	IL-17A/F, RORγt; FOXP3, IL-10; Th17/Treg ratio	IL-17/IL-23 pathway modulation; restoring Treg function	Human blood/tissue + experimental	Emerging	Likely stage-dependent; may require endotype stratification
IL-23/IL-17 inflammatory amplification	IL-23, IL-17A, downstream chemokines	IL-17 inhibitors/IL-23 blockade (largely indirect evidence in PBC)	Indirect clinical + experimental	Emerging/indirect	Clarify “indirect evidence”; prioritize biomarker-guided trials
CD8^+^ cytotoxicity/exhaustion	GZMB, PRF1, IFN-γ; PD-1, TIM-3, LAG-3	Checkpoint modulation; limiting cytotoxic injury	Human tissue + animal models	Emerging to preclinical	Balance efficacy against infection risk, impaired tumor immune surveillance, and off-target suppression of protective cytotoxic immunity; tissue context matters
Tfh–IL-21–B-cell axis	Tfh markers, IL-21; plasmablast frequency; AMA	B-cell/plasmablast targeting; disrupting Tfh–B interaction	Human studies + mechanistic	Emerging	Useful for endotypes with strong humoral signatures
B cells/antigen presentation	MHC-II, co-stimulation; antigen-specific responses	Modulating antigen presentation; B-cell-directed strategies	Human + experimental	Emerging	Needs careful patient selection and safety monitoring, including attention to infection risk and unintended systemic immunosuppression
APC-driven innate programs	DC/macrophage activation signatures; IL-12/23; TLR pathways	Innate immune modulation; cytokine pathway targeting	Human tissue + experimental	Emerging	May be more relevant in early/inflammatory stages
Mitochondrial stress/defective mitophagy–immune crosstalk	Mitochondrial stress signatures; candidate mtDAMPs; ROS	Mitophagy restoration; mitochondrial homeostasis support	Experimental + hypothesis	Preclinical/hypothesis-generating	Clearly separate established evidence vs. hypothesis; define unverified links
Bile acids/FXR and immune regulation	Bile acid profiles; FXR-related programs	FXR agonists; bile acid signaling modulation	Clinical + mechanistic	Relatively advanced/clinically linked	Bridge metabolism–immunity; potential combination strategies
Cholangiocyte senescence/ductular reaction	Senescence markers; SASP-related signals; ductular reaction features	Targeting senescence-associated pathways; tissue remodeling modulation	Human histology + emerging multi-omics	Emerging	Critical for chronic remodeling/fibrosis endotype
Unconventional T cells (γδT/MAIT/iNKT/DNT)	Subset frequency; cytokines; activation markers	Subset- or pathway-focused modulation (context-dependent)	Human + experimental	Preclinical/exploratory	Emphasize plasticity; broad targeting may suppress both pathogenic and tissue-protective functions, so interpretation should be microenvironment- and stage-dependent

This table summarizes the major immune pathways implicated in PBC, together with representative biomarkers/readouts, potential therapeutic angles, principal sources and levels of supporting evidence, relative translational maturity, and key practical considerations for endotype- and stage-oriented strategies. The table is intended to distinguish pathways that are relatively more mature and clinically linked from those that remain emerging, preclinical, or hypothesis-generating in PBC.

Abbreviations: PBC, primary biliary cholangitis; Treg, regulatory T cell; GZMB, granzyme B; PRF1, perforin 1; TIM-3, T-cell immunoglobulin and mucin-domain containing-3; LAG-3, lymphocyte-activation gene 3; Tfh, T follicular helper cell; MHC-II, major histocompatibility complex class II; AMA, anti-mitochondrial antibody; APC, antigen-presenting cell; DC, dendritic cell; mtDAMPs, mitochondrial damage-associated molecular patterns; ROS, reactive oxygen species; FXR, farnesoid X receptor; SASP, senescence-associated secretory phenotype; MAIT, mucosa-associated invariant T cells; iNKT, invariant natural killer T cells; DNT, double-negative T cell.

## 6 Conclusion

PBC is no longer considered to be merely an autoimmune cholestatic liver disease, instead it is regarded as a complicated, immune system driven cholangiopathy in which the imbalance of T cell subsets plays a pivotal role in regulation. Current evidence indicates that Th17/Treg imbalance, bile duct-reactive cytotoxic CD8^+^ T cells, and functionally reprogrammed unconventional T cell populations — particularly γδ T cells — act synergistically to initiate a self-perpetuating pathogenic cascade. This cascade reshapes the biliary inflammatory microenvironment, integrates impaired mitophagy with bile acid-farnesoid X receptor signaling, and ultimately sustains chronic small bile duct injury and progressive fibrotic remodeling.

At a mechanistic level, key pathways such as the IL-23/IL-17 axis, the PPARγ-NF-κB pathway, TCR signaling, and bile acid-FXR-NF-κB crosstalk form an integrative web linking immune cell phenotypes with organ-level pathology. In that context, immune biomarkers such as the peripheral Th17/Treg ratio or bile duct-reactive CD8^+^ T-cell clonotypes are emerging primarily as descriptive or mechanistically informative markers, whereas their predictive value and realistic clinical translatability still require further validation. In this context, the near-term priority is to identify which biomarkers are most suitable for immune-state description, which may aid prediction or treatment stratification, and which are sufficiently robust and feasible for eventual clinical implementation.

Meanwhile, progress in more directed forms of immunomodulation — including IL-17/IL-23- and RORγt-targeted approaches, CD8^+^ T-cell-directed strategies, BTK-related modulation of γδ T-cell responses, and tolerance-inducing interventions such as low-dose IL-2 and Imm TOR — provides a conceptual basis for future multitarget and potentially reversible immunomodulation in PBC, although most of these approaches remain preclinical, indirectly supported, or not yet validated directly in PBC. Importantly, these strategies differ substantially in translational maturity: bile acid/FXR-linked approaches are comparatively closer to clinical applicability, cytokine- and co-signaling-directed strategies remain emerging, whereas selective CD8^+^ T-cell targeting, BTK-related modulation of γδ T-cell responses, and several tolerance-restoring or combinatorial approaches are still largely preclinical or hypothesis-generating.

Looking forward, we argue that it is not more mechanistic insight which will be needed but instead integration. Integration efforts should focus on (i) linking human relevant models to high dimensional immune profiling; (ii) linking T cell pathways with cholestatic injury, mitochondrial stress, and the gut-liver axis; and (iii) integrating clinical phenotypes (e.g., clinical, immunological, and molecular) to actionable, patient-specific decisions. If it can be integrated in an overall precision immunology strategy, the combination might - based on UDCA and second line treatments - eventually relate individual PBC immune endotypes with respective target combinations as well as intensity of treatment, accelerating a transition away from empiric, population-based management toward personalized, immune-guided therapy.
